# Supplementary material for: Computational study on the catalytic control of endo/exo Diels-Alder reactions by cavity quantum vacuum fluctuations
Source: Nat Commun. 2023 May 13;14:2766. doi: 10.1038/s41467-023-38474-w (PMC10183045; doi:10.1038/s41467-023-38474-w)
Supplement: Supplementary file 1 — Supplementary Information [file 41467_2023_38474_MOESM1_ESM.pdf]

Supplementary Information Computational Study on the Catalytic Control of *endo/exo* Diels-Alder Reactions by Cavity Quantum Vacuum Fluctuations

Fabijan Pavošević,[1,*] Robert L. Smith,[1,2] and Angel Rubio[1,3,4,*]

[1] *Center for Computational Quantum Physics, Flatiron Institute, 162 5th Ave., New York, 10010 NY, USA*
[2] *Department of Chemistry, Virginia Tech, Blacksburg, Virginia 24061, U.S.A.*
[3] *Max Planck Institute for the Structure and Dynamics of Matter and Center for Free-Electron Laser Science & Department of Physics, Luruper Chaussee 149, 22761 Hamburg, Germany*
[4] *Nano-Bio Spectroscopy Group and European Theoretical Spectroscopy Facility (ETSF), Universidad del País Vasco (UPV/EHU), Av. Tolosa 72, 20018 San Sebastian, Spain*

* To whom correspondence should be addressed. E-mail: fpavosevic@gmail.com, angel.rubio@mpsd.mpg.de

I. SUPPLEMENTARY DISCUSSION 1

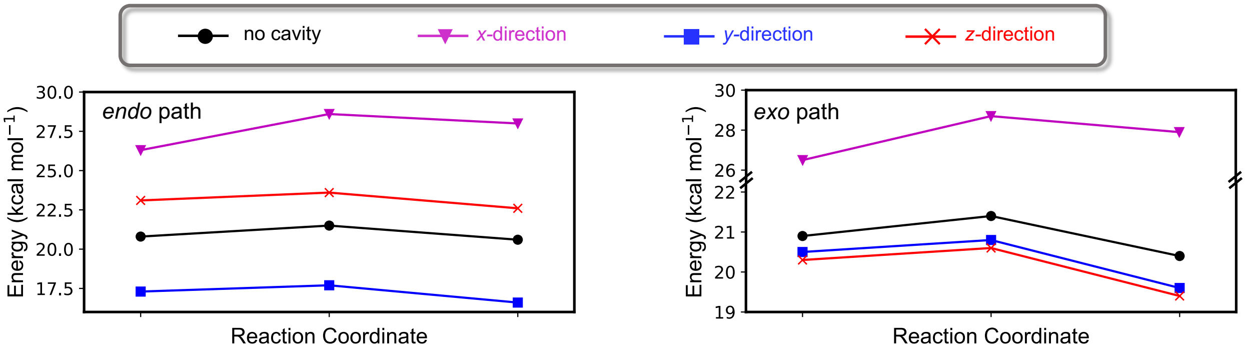

Supplementary Figure 1. **Effect of the strong light-matter interaction to transition state structures.** Reaction diagram around transition state of Diels–Alder cycloaddition reaction between cyclopentadiene (CPD) and acrylonitrile (AN) with respect to reaction coordinate for *endo* path (left panel) and *exo* path (right panel) calculated with the CCSD/cc-pVDZ (black) and QED-CCSD/cc-pVDZ methods. The quantum electrodynamics (QED) calculations employ the cavity frequency $\omega_{\text{cav}} = 1.5$ eV and light-matter coupling strength $|\lambda| = 0.1$ a.u. with one photon mode polarized in the x (magenta), y (blue), and z (red) molecular directions. Source data are provided as a Source Data file.

All of the reported calculations in this work assume that the molecular geometry does not change significantly due to strong light-matter interaction. This is however an approximation and in order to check its validity, we have performed the IRC calculation along the *endo* and *exo* paths at the MP2/cc-pVDZ level of theory that provided us with the path that connects the reaction complex and the product with the transition state. Although this path may not correspond to the CCSD or QED-CCSD path, but since reaction coordinate around the transition state mainly corresponds to the change in distance between CPD and AN, this is a good assumption. Next, we have taken one point from each side of the transition state and performed the CCSD and QED-CCSD calculations on these geometries as given in Supplementary Figure 1. The Supplementary Figure 1 shows that the maximum energy that is corresponding to the transition state is the same for both CCSD and QED-CCSD with all three light polarization directions. Based on that, we do not expect that the transition state will dramatically change inside the cavity for the selected value of the light-matter coupling. Additionally, because the calculations employ relatively large value of the light-matter coupling strength ($|\lambda| = 0.1$ a.u.), the changes in geometries with respect to the light-matter coupling strength will be less pronounced for the lower values of $|\lambda|$.

II. SUPPLEMENTARY DISCUSSION 2

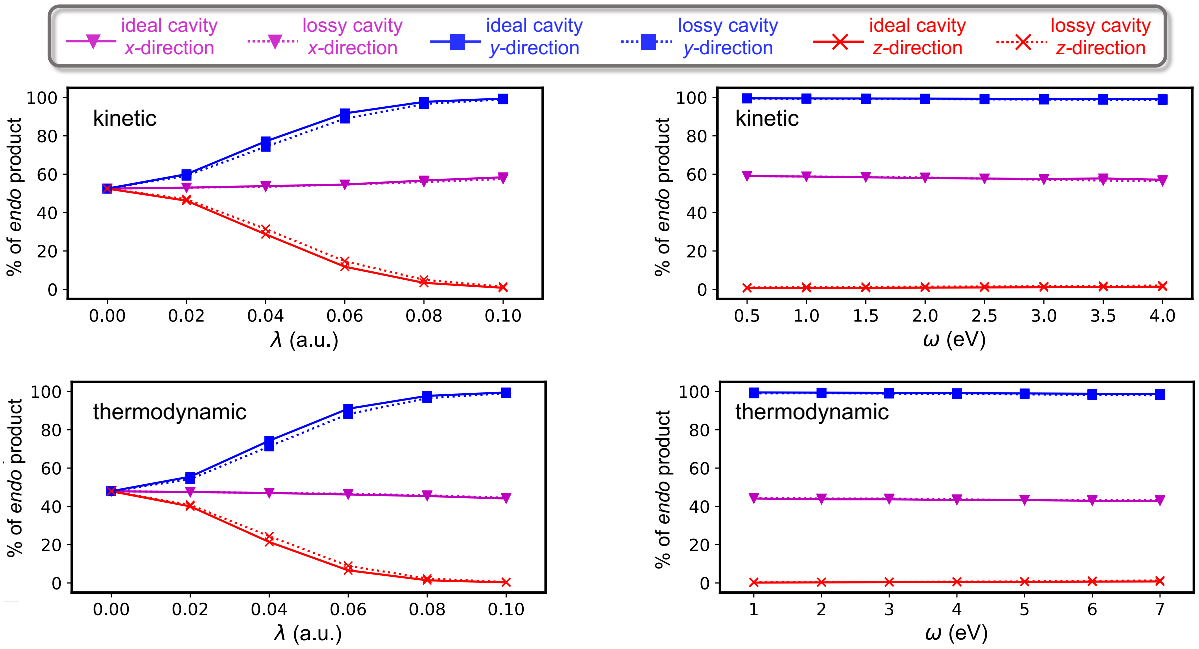

Supplementary Figure 2. Content of ***endo*** **product as a function of cavity parameters for reaction between cyclopentadiene (CPD) and acrylonitrile (AN).** Percentage of *endo* product for kinetically controlled (upper two panels) and thermodynamically controlled (lower two panels) Diels-Alder cycloaddition reaction between CPD and AN as a function of cavity coupling strength, λ, (left panels) and cavity frequency, ω, (right panels) calculated with the QED-CCSD method. The solid lines corresponds to ideal (lossless) cavity with one cavity mode polarized along the x (magenta), y (blue), and z (red) molecular directions. The dotted lines corresponds to the lossy (dissipative) cavity with 6,000 modes and dissipation constant 1 eV. The left panels are calculated for the cavity frequency 1.5 eV, whereas the right panels are calculated for the cavity coupling strength magnitude of 0.1 a.u. Source data are provided as a Source Data file.

The upper two panels of Supplementary Figure 2 shows the change in content of *endo* product for kinetically controlled Diels-Alder cycloaddition reaction between CPD and AN as the cavity light-matter coupling strength (left panel) and the cavity frequency (right panel) are increased. The lower two panels show the same process for the reaction under thermodynamic control. The content of *exo* product is calculated by subtracting the content of *endo* product from 100%. In the left two panels, the magnitude of the cavity coupling strength is increased from 0 a.u. to 0.1 a.u., while keeping the cavity frequency constant at 1.5 eV. When the cavity mode of an ideal cavity (solid lines) is polarized along the molecular x direction, the content of the *endo* product increases by a small amount, however, if the cavity mode is polarized along other two directions (y and z), the content of the *endo* product changes rapidly for both kinetically and thermodynamically controlled reactions. In the right two panels, the cavity frequency is increased from 1 eV to 7 eV, while keeping cavity coupling strength constant at 0.1 a.u. As evident from Supplementary Figure 2, the content of the *endo* product shows very little dependence on the cavity frequency for both kinetically and thermodynamically controlled reactions.

The upper and lower left panels of Supplementary Figure 3 shows the change in content of *endo* product for the Diels-Alder cycloaddition reaction between CPD and MeAN under kinetic and thermodynamic control, respectively, as the cavity light-matter coupling strength is increased from 0 a.u. to 0.1 a.u., while keeping the cavity frequency constant at 1.5 eV. In the case of ideal cavity with one cavity mode (solid lines) polarized along the molecular x direction, the content of *endo* product changes by a small amount as the coupling strength increases for reactions under kinetic and thermodynamic control. In the case of kinetically controlled reaction and when the cavity mode is polarized along the molecular y direction, the content of the *endo* product changes rapidly and reaching 46% of the *endo* product for $|\lambda| = 0.1$ a.u. In case of the thermodynamically controlled reaction, this change is even more pronounced, where the content of the *endo* product is 88% for $\lambda = 0.1$ a.u. As for the case when the cavity mode is polarized along the molecular z direction, the content of *exo* product becomes 99% at $\lambda = 0.1$ a.u. for both kinetically and thermodynamically controlled reactions. The right two panels of Supplementary Figure 3 show change in content of *endo* product as the cavity frequency is increased from 1 eV to 7 eV, while keeping cavity coupling strength

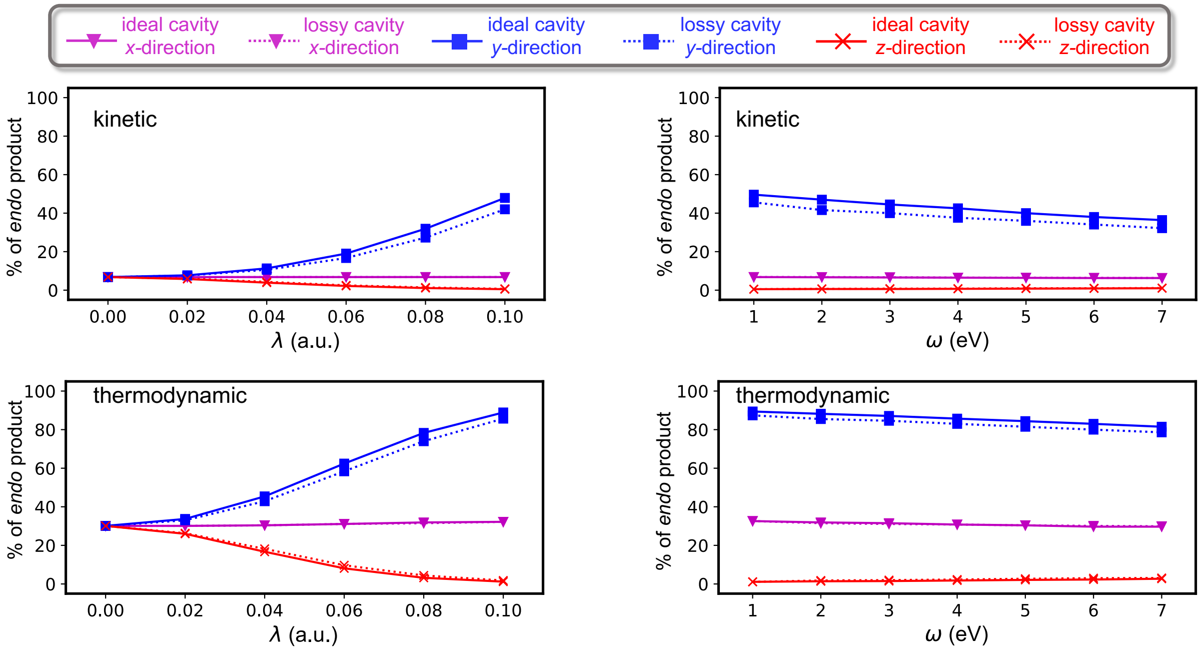

Supplementary Figure 3. **Content of** ***endo*** **product as a function of cavity parameters for reaction between cyclopentadiene (CPD) and methylacrylonitrile (MeAN).** Percentage of *endo* product for kinetically controlled (upper two panels) and thermodynamically controlled (lower two panels) Diels-Alder cycloaddition reaction between CPD and MeAN as a function of cavity coupling strength, λ, (left panels) and cavity frequency, ω, (right panels) calculated with the QED-CCSD method. The solid lines corresponds to ideal (lossless) cavity with one cavity mode polarized along the x (magenta), y (blue), and z (red) molecular directions. The dotted lines corresponds to the lossy (dissipative) cavity with 6,000 modes and dissipation constant 1 eV. The left panels are calculated for the cavity frequency 1.5 eV, whereas the right panels are calculated for the cavity coupling strength magnitude of 0.1 a.u. Source data are provided as a Source Data file.

constant at 0.1 a.u. As indicated in Supplementary Figure 3, for the case when the cavity modes are polarized along molecular x and z direction, the content of the *endo* product changes by a small amount for reaction under kinetic or thermodynamic control. In case when the cavity mode is polarized in the molecular y direction, the change in content of the *endo* product is much more pronounced as the cavity frequency changes under both kinetic and thermodynamic control.

Lastly, in Supplementary Figures 2 and 3, the dotted lines corresponds to the lossy (dissipative cavity) that is modeled with 6,000 modes and dissipation constant 1 eV. More information about the specifics of the calculations for the lossy cavity are provided below within the Supplementary Information. The results for the lossy cavity shown in Supplementary Figures 2 and 3 indicate that the cavity losses have only minor impact on the content of the *endo* product for the two investigated Diels-Alder cycloaddition reactions and under investigated conditions.

III. SUPPLEMENTARY DISCUSSION 3

Supplementary Table 1. **Calculated molecular dipole moments.** Dipole moment contributions (in a.u.) of reaction complexes, transition states, and products for Diels-Alder cycloaddition reactions of cyclopentadiene (CPD) with acrylonitrile (AN) and methylacrylonitrile (MeAN) calculated with the CCSD/cc-pVDZ method.

	CPD+AN					
	$\langle \mathbf{d} \rangle_x$		$\langle \mathbf{d} \rangle_y$		$\langle \mathbf{d} \rangle_z$	
	endo	*exo*	*endo*	*exo*	*endo*	*exo*
Reaction Complex	-0.164	-0.092	1.450	-0.945	-0.018	0.905
Transition State	-0.869	-0.765	1.232	-1.167	0.848	0.900
Product	-0.799	-0.738	1.053	-1.120	0.724	0.683

	CPD+MeAN					
	$\langle \mathbf{d} \rangle_x$		$\langle \mathbf{d} \rangle_y$		$\langle \mathbf{d} \rangle_z$	
	endo	*exo*	*endo*	*exo*	*endo*	*exo*
Reaction Complex	-0.082	0.036	1.426	-1.048	-0.261	0.753
Transition State	-0.674	-0.603	1.310	-1.191	0.765	0.911
Product	-0.674	-0.611	1.163	-1.231	0.618	0.590

Supplementary Table 1 shows molecular dipole moment contributions of reaction complexes, transition states, and products that occur along the reaction *endo* and *exo* paths in both studied reactions calculated with the CCSD/cc-pVDZ method. The total dipole moment of all molecular structures are mostly aligned with the direction of the nitrile ($-$CN) substituent, however, the orientation of the molecular dipole moment changes along the reaction path. Therefore, due to different orientations of the molecular dipole moment, different stationary structures along the reaction path experiences different coupling with the light. The component of the dipole moments along the x direction for all of the corresponding structures along the *endo* and *exo* paths are of roughly the same magnitude. However, the magnitude of the dipole moment component along the y direction for the reaction complex is $\sim$50% larger along the *endo* path than along the *exo* path. As a result, due to orientation of the nitrile group, the reaction complex along the *endo* path experiences much stronger coupling with the light. Similarly, the magnitude of the dipole moment component along the z direction for the reaction complex is much greater along the *exo* path than along the *endo* path, therefore the reaction complex along the *exo* path experiences much stronger coupling to the light.

IV. SUPPLEMENTARY DISCUSSION 4

Supplementary Table 2. **Energy contributions breakdown.** Energy contributions breakdown of reaction energy barrier (TS)[a] and reaction energy (ΔE)[b] (in kcal mol^{-1}) for Diels-Alder cycloaddition reactions of cyclopentadiene (CPD) with acrylonitrile (AN) and methylacrylonitrile (MeAN) calculated with QED-CCSD. The calculations employ the light-matter coupling strength of $|\lambda| = 0.1$ a.u. and one photon mode with frequency $\omega = 1.5$ eV.

	CPD+AN							
	no cavity		x-direction		y-direction		z-direction	
TS	*endo*	*exo*	*endo*	*exo*	*endo*	*exo*	*endo*	*exo*
electronic	20.8	20.9	23.3	23.3	20.0	21.3	21.2	20.5
dipole self energy	0.0	0.0	3.8	4.0	-3.1	-1.0	2.1	-0.3
dipolar coupling	0.0	0.0	-0.8	-0.8	0.4	0.1	-0.2	0.1
total[c]	20.8	20.9	26.3	26.5	17.3	20.4	23.1	20.3
ΔE	*endo*	*exo*	*endo*	*exo*	*endo*	*exo*	*endo*	*exo*
electronic	-29.6	-29.6	-27.5	-27.6	-30.5	-29.4	-29.6	-30.6
dipole self energy	0.0	0.0	1.9	1.8	-6.9	-4.6	-0.7	-3.6
dipolar coupling	0.0	0.0	-0.7	-0.7	0.7	0.4	-0.1	0.3
total[c]	-29.6	-29.6	-26.3	-26.5	-36.7	-33.6	-30.4	-33.9

	CPD+MeAN							
	no cavity		x-direction		y-direction		z-direction	
TS	*endo*	*exo*	*endo*	*exo*	*endo*	*exo*	*endo*	*exo*
electronic	23.6	22.1	26.5	24.9	23.6	22.4	23.3	21.7
dipole self energy	0.0	0.0	3.2	3.2	-2.4	-1.1	1.6	-0.2
dipolar coupling	0.0	0.0	-0.7	-0.7	0.3	0.1	-0.2	0.1
total[c]	23.6	22.1	29.0	27.4	21.5	21.4	24.7	21.6
ΔE	*endo*	*exo*	*endo*	*exo*	*endo*	*exo*	*endo*	*exo*
electronic	-27.1	-27.6	-24.9	-25.3	-27.3	-27.4	-28.0	-28.7
dipole self energy	0.0	0.0	1.2	1.2	-6.0	-4.5	-1.5	-3.7
dipolar coupling	0.0	0.0	-0.6	-0.7	0.6	0.4	0.0	0.3
total[c]	-27.1	-27.6	-24.3	-24.8	-32.7	-31.5	-29.5	-32.1

[a]Calculated as the energy difference between the transition state and the reaction complex.
[b]Calculated as the energy difference between the product and the reaction complex.
[c]Defined as the sum of electronic, dipole self energy, and dipolar coupling.

Supplementary Table 2 shows the energy contributions breakdown for the two studied Diels-Alder cycloaddition reactions. The energy contributions for the QED-CCSD method with one cavity mode are: electronic $\left(\langle 0^{\mathrm{e}}0^{\mathrm{ph}}|\hat{H}^{\mathrm{e}}|0^{\mathrm{e}}0^{\mathrm{ph}}\rangle + \bar{g}_{ij}^{ab} \times (0.25 \cdot t_{ab}^{ij} + 0.5 \cdot t_a^i t_b^j)\right)$, dipole self-energy $\left(\langle 0^{\mathrm{e}}0^{\mathrm{ph}}|\frac{1}{2}(\boldsymbol{\lambda} \cdot \Delta\boldsymbol{d})^2|0^{\mathrm{e}}0^{\mathrm{ph}}\rangle + ((\boldsymbol{\lambda} \cdot \boldsymbol{d})^2)_{ij}^{ab} \times (0.25 \cdot t_{ab}^{ij} + 0.5 \cdot t_a^i t_b^j)\right)$, and dipolar coupling $\left((\boldsymbol{\lambda}\cdot\boldsymbol{d})_i^a \times (t_{a,1}^i + t_a^i t_1)\right)$. The $\langle 0^{\mathrm{e}}0^{\mathrm{ph}}|\hat{H}^{\mathrm{e}}|0^{\mathrm{e}}0^{\mathrm{ph}}\rangle$ and $\langle 0^{\mathrm{e}}0^{\mathrm{ph}}|\frac{1}{2}(\boldsymbol{\lambda}\cdot\Delta\boldsymbol{d})^2|0^{\mathrm{e}}0^{\mathrm{ph}}\rangle$ corresponds to electronic and dipole self-energy contributions, respectively, calculated with the QED-Hartree-Fock method. The calculations employ cavity parameters $\lambda = 0.1$ a.u. and $\omega = 1.5$ eV. The Supplementary Table 2 indicate that the electronic energy contributes the most to the energy, whereas the changes in energy due to the cavity are mainly due to the dipole self-energy contributions. These changes in the dipole self-energy are due to the quantum vacuum fluctuations that are the main driving forces for the reactions inside a cavity.

V. SUPPLEMENTARY DISCUSSION 5

In the case of ideal cavity, only one discrete sharp photon mode is used with cavity coupling strength $\boldsymbol{\lambda}$. However, to account for cavity losses, we have employed the theoretical framework introduced in Ref. [1], in which the photon mode is broaden by explicitly including multiple discrete photon modes. As a result, the values of $\boldsymbol{\lambda}_\alpha$, where α corresponds to α-th photon mode, are sampled from a Lorentzian distribution as

$$|\boldsymbol{\lambda}_\alpha|^2 = |\boldsymbol{\lambda}|^2 L(\Delta\omega, \kappa, \omega_\alpha, \omega_c), \tag{1}$$

where $L(\Delta\omega, \kappa, \omega_\alpha, \omega_c)$ is defined as

$$L(\Delta\omega, \kappa, \omega_\alpha, \omega_c) = \Delta\omega \frac{1}{2\pi} \frac{\kappa}{(\omega_\alpha - \omega_c)^2 + (\kappa/2)^2}. \tag{2}$$

In this equation, ω_c is the (central) frequency around which the distribution is centered, $\Delta\omega$ is a constant frequency spacing, and κ is dissipation constant that accounts for cavity losses. The dissipation constant controls the broadening of the Lorentzian distribution, therefore for $\kappa = 0$ eV, the result for the lossless cavity is recovered.

In this work, we have sampled $\boldsymbol{\lambda}_\alpha$ with central frequency of $\omega = 1.5$ eV and $\boldsymbol{\lambda} = 1$ a.u. in the indicated polarization direction with three different values of dissipation constant $\kappa = 0.01$ eV, 0.1 eV, 1 eV. Moreover, the $\Delta\omega = 0.001$ eV and the frequencies ω_α range from 0 eV to 6 eV. This results in the QED-CCSD calculation with 6,000 cavity modes. The results for different values of dissipation constant κ are given in Supplementary Table 3 for the two different Diels-Alder cycloaddition reactions along *endo* and *exo* paths. As shown in Supplementary Table 3, the overall results do not change significantly with increase of κ.

Supplementary Table 3. **Dissipative cavity calculations.** Change of reaction energy barrier (TS)[a] and reaction energy (ΔE)[b] (in kcal mol^{-1}) with respect to dissipation constant κ for two Diels-Alder cycloaddition reactions of cyclopentadiene (CPD) with acrylonitrile (AN) and methylacrylonitrile (MeAN). The calculations employ the light-matter coupling strength of $|\lambda| = 0.1$ a.u. with central frequency $\omega_c = 1.5$ eV.

		CPD+AN					
		x-direction		y-direction		z-direction	
		endo	*exo*	*endo*	*exo*	*endo*	*exo*
$\kappa = 0.0$ eV	TS	26.3	26.5	17.3	20.4	23.1	20.3
	ΔE	-26.3	-26.5	-36.7	-33.6	-30.4	-33.9
$\kappa = 0.01$ eV	TS	26.3	26.5	17.3	20.4	23.1	20.3
	ΔE	-26.3	-26.5	-36.7	-33.6	-30.4	-33.9
$\kappa = 0.1$ eV	TS	26.3	26.5	17.4	20.4	23.1	20.3
	ΔE	-26.4	-26.5	-26.7	-33.6	-30.4	-33.9
$\kappa = 1$ eV	TS	25.9	26.0	17.6	20.5	22.8	20.3
	ΔE	-26.7	-26.8	-35.9	-33.2	-30.3	-33.4
		CPD+MeAN					
		x-direction		y-direction		z-direction	
		endo	*exo*	*endo*	*exo*	*endo*	*exo*
$\kappa = 0.0$ eV	TS	29.0	27.4	21.5	21.4	24.7	21.6
	ΔE	-24.3	-24.8	-32.7	-31.5	-29.5	-32.1
$\kappa = 0.01$ eV	TS	29.0	27.4	21.5	21.4	24.7	21.6
	ΔE	-24.3	-24.8	-32.7	-31.5	-29.5	-32.1
$\kappa = 0.1$ eV	TS	28.9	27.4	21.5	21.4	24.7	21.6
	ΔE	-24.3	-24.8	-32.7	-31.5	-29.5	-32.0
$\kappa = 1$ eV	TS	28.5	26.9	21.7	21.5	24.5	21.6
	ΔE	-24.5	-25.0	-32.1	-31.1	-29.2	-31.6

[a]Calculated as the energy difference between the transition state and the reaction complex.
[b]Calculated as the energy difference between the product and the reaction complex.

VI. SUPPLEMENTARY DISCUSSION 6

Supplementary Table 4. **Benchmarking the QED-CCSD method.** Reaction energy barrier (TS)[a] and reaction energy (ΔE)[b] (in kcal mol^{-1}) for Diels-Alder cycloaddition reaction between cyclopentadiene (CPD) and acrylonitrile (AN) calculated with QED-CCSD/cc-pVDZ and QED-CCSD-22/cc-pVDZ methods. Employed cavity parameters are $|\lambda| = 0.1$ a.u. and with one photon mode of frequency $\omega = 1.5$ eV.

	QED-CCSD		QED-CCSD-22	
endo-path	TS	ΔE	TS	ΔE
x-direction	26.3	-26.3	25.6	-26.7
y-direction	17.3	-36.7	17.5	-36.3
z-direction	23.1	-30.4	22.8	-30.4
	QED-CCSD		QED-CCSD-22	
exo-path	TS	ΔE	TS	ΔE
x-direction	26.5	-26.5	25.8	-26.8
y-direction	20.4	-33.6	20.3	-33.5
z-direction	20.3	-33.9	20.2	-33.7

[a]Calculated as the energy difference between the transition state and the reaction complex.
[b]Calculated as the energy difference between the product and the reaction complex.

As discussed in the main paper, the employed QED-CCSD method is obtained from the QED-CCSD-22 method by discarding the $\frac{1}{4}\sum_{\alpha} t^{ij}_{ab,\alpha} a^{ab}_{ij} b^{\dagger}_{\alpha}$, $\frac{1}{2}\sum_{\alpha\beta} t_{\alpha\beta} b^{\dagger}_{\alpha} b^{\dagger}_{\beta}$, $\frac{1}{2}\sum_{\alpha\beta} t^{i}_{a,\alpha\beta} a^{a}_{i} b^{\dagger}_{\alpha} b^{\dagger}_{\beta}$, and $\frac{1}{8}\sum_{\alpha\beta} t^{ij}_{ab,\alpha\beta} a^{ab}_{ij} b^{\dagger}_{\alpha} b^{\dagger}_{\beta}$ terms from the $\hat{T}$ cluster operator. As shown previously [2], the QED-CCSD-22 method provides an accurate results for the systems confined to a cavity and therefore it is used here as a benchmark. Supplementary Table 4 shows the reaction energy barriers and reaction energies for the Diels-Alder cycloaddition reaction between cyclopentadiene (CPD) and acrylonitrile (AN) calculated with the QED-CCSD and QED-CCSD-22 methods. For the case when the cavity mode is polarized along the molecular x-direction, the calculated reaction energy barriers (TS) using the QED-CCSD method deviates by 0.7 kcal mol^{-1} from the ones obtained with the QED-CCSD-22 method, whereas in the case of calculated reaction energy (ΔE), the deviation is $\sim$0.4 kcal mol^{-1}. With regard to cavity polarization in the molecular y-direction and the molecular z-direction, the discrepancy between the two investigated methods is below $\sim$0.4 kcal mol^{-1} for both energy barrier (TS) and reaction energy (ΔE). Because the calculated discrepancies between the two methods do not exceed the chemical accuracy of 1 kcal mol^{-1}, the QED-CCSD method is accurate for the purposes of this work.

VII. SUPPLEMENTARY REFERENCES

[1] D. S. Wang, T. Neuman, J. Flick, and P. Narang, J. Chem. Phys. **154**, 104109 (2021).
[2] F. Pavošević, S. Hammes-Schiffer, A. Rubio, and J. Flick, J. Am. Chem. Soc. **144**, 4995–5002 (2022).
